# Round the Hospitals

**Published:** 1919-09-13

**Authors:** 


### ROUND THE HOSPITALS.

The growth of the Local Centres of the College of Nursing is one of those unmistakable signs of vigorous life which afford occasion for justifiable pride and satisfaction. At the beginning of this new session they are found to number twenty, situated as follows:—Aberdeen, Birmingham, Brighton and Hove, Bristol, Cambridge, Cornwall, Derby, Edinburgh, Glasgow, Yorkshire (Leeds), Leicester, Lincoln, Liverpool, London, Manchester, Norfolk and Norwich, Northumberland and Durham, Nottingham, Plymouth, and Sheffield. The position with regard to these centres is that in every one post-graduate courses of lectures are being arranged. One centre, Brighton and Hove, has already a flourishing residential club. Another, Liverpool, has money in hand for forming a club and is only forced to delay owing to the difficulty of finding a central site or suitable premises. In

**ROUND THE HOSPITALS—(*continued*).**

Manchester, Birmingham, and Leeds the members are pursuing a vigorous forward policy with a view to securing quarters as speedily as possible. In all the centres periodic meetings and discussions keep the interest of the members in the College and in nursing affairs generally alive and keen. In all the work of strengthening the hands of the authorities at headquarters continues to progress.

The various centres of the College, although entirely independent one of another, are by no means selfishly self-centred. They exchange speakers and are mutually helpful. Moreover, they combine for aims stretching out beyond their own boundaries. The great desire at the moment among the College members is to build up an endowment for a Chair of Nursing at King's College for Women, where already there are some twelve or fourteen nursing sisters taking the special course which has been arranged for their benefit. All the centres have promised financial help towards this admirable scheme, and in several the members have pledged themselves to contribute far more than was originally suggested. It is undoubtedly a project which must mature slowly, but its prospects are already bright.

No one will grudge the extra ten million a year which increased pay to officers and men has added to the Navy estimates. Indeed, we could have borne with equanimity to see the estimates still further raised to cover the small cost of increasing the salaries of the naval nurses. They are no higher than they were before the war, while prices have advanced some 100 per cent. Now that the question of gratuities has been settled, it is time that the Admiralty considered the question of re-organising the whole of the naval nursing service. Rightly or wrongly, there is an impression that it would be for the advantage of Q.A.R.N.N.S. if the head sister were raised to the status of matron-in-chief, with the same powers as attach to this post in the sister service.

The proposed scheme for preparing a "National Roll of the Great War" involves vast expenditure. To provide a permanent record of all the men and women who fought or worked for the Empire in various capacities during the war with a register of names and honours, together with brief biographies, is an enterprise of such dimensions as staggers the imagination. Every branch of the nursing service is to be included, as well as the supplementary women's Army, Navy, and Air services. Even munition workers are to find a place in this extraordinary production, and a claim is being made for the inclusion also of women who took up land work. We are not very hopeful about its success. But it is a field where fraudulent imitators are peculiarly likely to attempt a harvest. We would therefore strongly caution our readers not to pay any money in advance for copies of any volume containing their names or deeds, and to turn a deaf ear to any plea, however plausible, for subscriptions to similar enterprises.

The referendum of the M.A.B. nursing staff on the question of hours and holidays has ended rather unexpectedly. Approximately 800 nurses voted for forty-eight hours a week and holidays as they were, while some 600 only voted for fifty hours and a fortnight's holiday twice a year. It is rather curious to notice that the nurses voted mainly collectively, whole institutions recording their opinion to the same effect. It can be understood that nurses whose homes lie at a distance from their place of work would prefer a longer annual holiday to leave twice a year, but except from this point of view the fifty-hour week works out as distinctly more advantageous to the nurses.

The institutions of the Metropolitan Asylums Board are among those which suffered most severely during the war. Some of their best nurses, to the number of 156, volunteered for war service, and by no means all of these have returned to their posts now they are set free. Moreover, the class of worker who used to make good probationers in fever and children's institutions was lured away to forms of war work offering higher pay and more freedom. In spite of handsome alterations in pay and hours which contribute to make the M.A.B. service really attractive, the shortage of probationers continues, and it appears doubtful whether these alterations have yet penetrated through to the knowledge of the women who are wanted. There is a great gulf between the institution world and the public. The secret of attracting probationers lies in bridging it, and unless that can be done girls will continue to drift away into less desirable occupations.

The movement in favour of securing better pensions for Army sisters has the approval of all who have taken the trouble to master the intricacies of the regulations. Under quite ordinary circumstances the pension of an Army sister retiring at the age of 55 will amount to no more than 17s. 6d. a week, and few will contend that this is an adequate sum. Miss Gertrude Vaughan, writing in the *Daily Telegraph*, urges that pensions should be based not, as at present, on pay, but on pay plus emoluments and allowances. Viscount Peel recently announced that a committee was considering the present rates of retired pay of members of Q.A.I.M.N.S. and a pronouncement on the subject may be expected in due course. It is very hard on women whose emoluments are actually of higher value than their pay that these should be left out of sight entirely when calculating pensions. Whatever increase the Committee may recommend we trust it will be made retrospective, so that the lot of former Army sisters at present attempting to live on their pittance may be alleviated.

It is a very satisfactory matter that military nurses are no longer left entirely unaided in the struggle to resume their place in the Civil Nursing Service. By filling in a form of application (D. 66) for the Out-of-Work Donation Policy of a Civilian Worker, a form which can be obtained at the nearest

### ROUND THE HOSPITALS—(*continued*).

Labour Exchange, the nurse who fulfils the conditions may obtain a weekly allowance of 15s. for thirteen weeks, or until she obtains employment. The sum is not large, but it makes a considerable difference to women anxiously watching the ebb of previous savings. The demobilisation of so many nurses could hardly have fallen out at a more disadvantageous time than in the summer, when, for various causes, employment is always slackest among nurses. With the autumn season a distinct improvement may be expected. Private staffs are replenished, alterations and enlargements commence in institution staffs, and there is general movement all along the line. It is a safe conjecture that at the end of the thirteen weeks there will be few nurses in receipt of unemployment benefit.

---

In many instances the military authorities have left the Poor Law hospitals which they took over from the authorities in better condition than they found them. At Richmond, for instance, an excellent operating-room was added with all necessary appliances, as well as a side chapel and a mortuary table. True to the traditions of Bumbledom, the House Committee recommended that the operating-room should not be retained. Happily, saner counsels prevailed. The Mayor took a public-spirited view of the matter and urged the guardians to look to the future when their institution would become a citizens' hospital and the theatre would be a necessity. Lady Nott Bower, who seconded the amendment for retaining the operating-room, wisely emphasised the need for developing a training-school for nurses in the institution. And in the end, Richmond retains the improvements brought to its doors by the fortune of war.

---

The little pamphlet which gives an account of the Millicent Sutherland Ambulance ought to be widely read, for it throws much light on the heroic efforts of the nursing sisters during the two years preceding the signing of the Armistice. The Ambulance was at first situated on the sand dunes in the neighbourhood of Calais. It was then transferred to a site near St. Omer, so situated that it was able to receive patients direct from the dressing-stations. In the great attack of the Allies the ambulance moved gradually forward, first to Hazebrouck, and then to Roubaix. In the four years preceding the Armistice 440 British officers, 5,414 British N.C.O.s and men, about 2,000 French and other Allies were treated. The balance in hand—£4,614—is being devoted to alleviate distress in the devastated regions, and the Duchess of Sutherland has made a grant besides from the funds to the North Staffs Cripples' Guild. Moreover, certain old patients of the Ambulance are to be relieved in their disablement. It is a fine record of patriotic effort, nobly conceived and carried out in all its details with devotion and brains.

---

We are not to forget the needs of the suffering peasantry in the country which has so long been familiar to our minds as "the Front." The French Red Cross Society believes nothing will so well ensure actively sympathetic remembrance as a personal view of the wretched havoc wrought, and is arranging a series of conducted tours through the region. The President of the Royal Institute of British Architects, the Sheriff of London, and other influential people have started on a visit to the devastated areas. Much can be done from this country to alleviate the distress of the returning population and aid in the stupendous task of reconstruction, and for this publicity is needed.

---

Many of our readers will be glad to hear of the winter course of Elementary Lectures on Infant Care arranged by the National Association for the Prevention of Infant Mortality and for the Welfare of Infancy. Under its somewhat unwieldy title the Society is doing excellent work on Infant Care. The present course of lectures is to be given on Mondays from 5.30 to 6.30 at 1 Wimpole Street, W. 1. The charge for the twelve lectures is 5s., and for single lectures 1s., tickets to be obtained in advance from the Secretary, 4 Tavistock Square, W.C. 1. The opening lecture on September 29 is on Ante-Natal Hygiene, by Lady Barrett, C.B.E., M.D., M.S., Physician Diseases of Women, Royal Free Hospital. We are glad to note that prevention plays a large part in the course. The lectures on the early recognition, causes, and prevention of infectious diseases, ear and throat troubles, and defects of eyesight should be particularly helpful.

---

Women of the working classes have taken very kindly to the maternity homes now springing up in all directions, and it is likely they will still further increase and develop. The latest to be opened is one in Ravenscourt Park, provided by the Hammersmith Borough Council. It has accommodation for twenty maternity cases, and a ward has been added with twelve cots for children. The cost will be 30s. for a fortnight's treatment. We are glad to learn that the new institution is to be a training-school for midwives. We trust great care will be taken from the very first to protect pupil-midwives from the conditions of excessive work and worry which attend the intensive courses of instruction in many lying-in establishments.

---

The Bedford County Hospital staff has decided to form a Past and Present Nurses League for the purpose of linking together all who are at present working in the hospital with those who have passed from its walls to other work. The League aims at maintaining the highest possible standard of nursing at the hospital, and it will form a register of members. One rather novel and interesting department of the League activities is to be the development to the fullest extent of the means placed at the disposal of the staff for mental and physical recreation. For lack of hearty stimulus from within, nurses often grow very slack in using their

**ROUND THE HOSPITALS**—(*continued*).

opportunities, and we believe the League, even from this point of view alone, has a future before it. The meeting which constituted the League was presided over by the Chairman of the Board of Management, and the interest displayed by the nurses, who attended in large numbers, augurs well for the success of the undertaking. Honorary members subscribe 10s. a year, and nurse members 5s. There is to be a League journal, but it will require skilful handling if it is not to be a heavy charge on the funds.

The trial of Quien, accused of betraying to the Germans Miss Cavell's rescue of British, Belgian, and French soldiers, has ended with a verdict of guilty, coupled with a death sentence. But six of the judges have recommended him to mercy, and he has lodged an appeal, so that more will yet be heard of this notorious individual. In the end, his dealings in respect of Miss Cavell proved to be so obscure that no actual proof of his complicity in the black deed of denouncing her to the Germans was forthcoming, though evidence of his activities as a German spy lent probability to the accusations against him of receiving shelter and benefits from her in order to betray her. The trial, which has excited prodigious interest in Paris, was chiefly noteworthy from an English point of view from the side-lights it threw on Miss Cavell's life and character. The story of her dealings with the maid Pauline, the evidence of Miss Elizabeth Wilkins, one of her nursing staff, the constant references by witnesses to the reverence in which the martyred nurse was held, even by the Germans, make up a new chapter in her life. Miss Wilkins took charge when Miss Cavell was shot and Miss Cavell wrote to her: "I am more worried about the school than about my own fate." In her last letter thanking the nurses for all they had done for her during her imprisonment, she ended: "I am not afraid, but quite happy." That is the final impression left on the mind after reading and re-reading the tangled evidence of this notable trial. Whatever Quien may have done or attempted, he was powerless to trouble the serene depths of a mind at one with God.

We watch with amazement the go-ahead tactics of nursing reformers in the Antipodes. The Minister of Public Health in Wellington, New Zealand, was approached in July by a deputation from the Industries Committee, who represented that the pay of probationers was altogether inadequate. They urged that £1 a week should be paid for the first year, with yearly increase, and that staff-nurses who at present receive £80 to £100 a year should receive a large addition to their salaries. Making all allowance for the difference in the purchasing power of money between England and New Zealand the fact remains that probationers receive free training, board, lodging, and laundry expenses, and that there is no profession of equal status with that of nursing which provides such extensive benefits for its students or apprentices free of all cost, in addition to a fair commencing salary. If probationers' pay were reckoned at its true value plus emoluments we should hear less about their being under-paid.

The Ranyard Nurses' Committee lay great stress in their report for 1918 on the fact that there are still in Greater London many districts where even in normal times no nursing help is yet available. Both in Balham and Lewisham the needs are urgent, but it is satisfactory to learn that a beginning is being made in both these crowded areas, and that in Brockley and Crofton Park a provident scheme has been organised. The Committee announce that the equipment and maintenance of a nurse costs, roughly, £130 a year. The nurses are provided for the sick poor without payment, and how well this rule is observed may be seen by the negligible amount (under £25) contributed under the head of patients' payments. It is a matter for earnest consideration whether the London "poor" are not fully as well able to pay 4s. a year for the benefit of skilled nursing as the agricultural labourer. The provident system has taken deep root in all parts of the Kingdom, and we believe could be readily extended to the towns, where working men already begin to prefer a contributory basis to a charitable one for medical aid. The Ranyard Nurses were long pioneers in nursing matters, and their work is priceless. They must not be suffered to lag behind in modern days through the maintenance of a principle which may come before long to be recognised as a blunder.

Managers of voluntary institutions where nurses, owing to an increase of staff, are crowded out of the nurses' home might take a hint from the Watford Guardians and adapt Army huts to accommodate some of the nurses until building prospects improve. Two huts at a cost of £55 each have been purchased to supplement the accommodation at this Poor Law infirmary. If the alterations are carried out intelligently we believe the result will be more satisfactory and far less costly than housing nurses out in lodgings.

By the recent closing down of Percy House, Isleworth, one of the largest Red Cross hospitals in the country, the Brentford Guardians come into possession of their own again, largely improved and adapted to modern medical and surgical requirements through private generosity. As a military hospital it contained 283 beds, and nearly 5,000 soldiers, principally from overseas, were nursed there, with but one death. It is earnestly to be hoped that the Guardians will take full advantage of the alterations, and that in particular the operating-theatre and other special departments may be retained, as at Richmond, with a view to the expansion of the institution into a municipal hospital in the near future.

